# Host-Guest Interaction between Herbicide Oxadiargyl and Hydroxypropyl-****β****-Cyclodextrin

**DOI:** 10.1155/2013/825206

**Published:** 2013-11-25

**Authors:** Sofia Benfeito, Tiago Rodrigues, Jorge Garrido, Fernanda Borges, E. Manuela Garrido

**Affiliations:** ^1^Departamento de Engenharia Química, Instituto Superior de Engenharia do Porto (ISEP), Instituto Politécnico do Porto, 4200-072 Porto, Portugal; ^2^CIQ/Departamento de Química e Bioquímica, Faculdade de Ciências, Universidade do Porto, 4169-007 Porto, Portugal

## Abstract

In the face of a growing human population and increased urbanization, the demand for pesticides will simply rise. Farmers must escalate yields on increasingly fewer farm acres. However, the risks of pesticides, whether real or perceived, may force changes in the way these chemicals are used. Scientists are working toward pest control plans that are environmentally sound, effective, and profitable. In this context the development of new pesticide formulations which may improve application effectiveness, safety, handling, and storage can be pointed out as a solution. As a contribution to the area, the microencapsulation of the herbicide oxadiargyl (OXA) in (2-hydroxypropyl)-**β**-cyclodextrin (HP-**β**-CD) was performed. The study was conducted in different aqueous media (ultrapure water and in different pH buffer solutions). In all cases an increment of the oxadiargyl solubility as a function of the HP-**β**-CD concentration that has been related to the formation of an inclusion complex was verified. UV-Vis and NMR experiments allowed concluding that the stoichiometry of the OXA/HP-**β**-CD complex formed is 1 : 1. The gathered results can be regarded as an important step for its removal from industrial effluents and/or to increase the stabilizing action, encapsulation, and adsorption in water treatment plants.

## 1. Introduction

The use of chemicals in agriculture is vital to control the destruction of crops by pests. These practices often involve an excessive amount of pesticides, causing great environmental problems. 

Currently there is a growing concern about environmental protection and reducing the environmental impact of pesticides. One of the approaches aiming to minimize their harmful effects is considered to be the application of nanotechnology solutions [1–3]. In fact, they can increase pesticides efficiency and then can reduce their negative impact on the environment and the harmful effects associated with the contact by workers who apply these substances. In the last decades, the molecular complexation into nanomaterials has been considered as a successful area to improve selectivity, separation, and solubilization of various active chemicals. Although numerous complexing agents have been reported to be effective for the purpose, such as fullerenes, metal oxides nanoparticles, or mesoporous silica matrix, they have been considered to be costly to synthesize and to be with limited capabilities [1–3]. The restrictions are mainly related to their low solubility and toxicity for environment and human health [4, 5].

However nanomaterials based on polymeric groups, such as cyclodextrins (CDs), have been up to date pointed out as the best choice to encapsulate bioactive molecules. Cyclodextrins (CDs) are cyclic biodegradable oligosaccharides formed by six to eight D-glucose units linked by *α*-1,4-glucose bonds considered to be interesting organic host systems as they have a hydrophobic inner cavity available to form noncovalent inclusion complexes with a wide variety of organic molecules of appropriate shape and size. In addition, CDs**  **have high aqueous solubility, declared to be nontoxic, and their structures are rigid and well defined [6–8]. For these reasons, CDs have attracted considerable attention in pesticide field as the physicochemical properties of the guest can be notably changed upon complexation.

Actually, the significant improvements in cyclodextrins production, coupled with low production costs, permit their application in agriculture. Encapsulation of pesticides by CDs can enhance their efficacy by increasing the dissolution rate and chemical and thermal stability and reducing their volatility as well as their bioavailability for biodegradation [9–13].

Oxadiargyl  (5-tert-butyl-3-[2, 4-dichloro-5-(prop-2-ynyloxy) phenyl]-1,3,4-oxadiazol-2(3*H*)-one) is an herbicide developed for the control of annual grasses, sedges, and broad-leaved weeds in rice [14]. However, its low aqueous solubility led to a facile accumulation in water or soil and to a rapid poisoning of environment.

**Figure 1:**
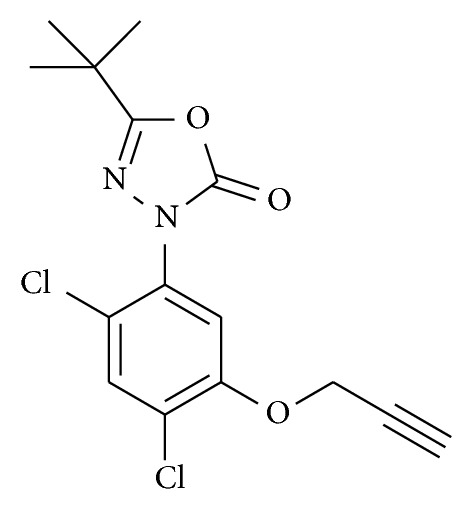
Molecular structure of the herbicide oxadiargyl.

Continuing our investigation on the complexation of pesticides with cyclodextrins herein, we report the inclusion studies of the herbicide oxadiargyl (OXA) (Figure 1) with (2-hydroxypropyl)-*β*-cyclodextrin (HP-*β*-CD) in different aqueous media. Ultraviolet spectroscopy studies have been used to attain the stoichiometry and binding constants of the inclusion complex. Nuclear magnetic resonance (NMR) studies have been carried out to elucidate the strength and binding mode of association of the complex.

## 2. Experimental

### 2.1. Chemicals

Oxadiargyl (OXA) and (2-hydroxypropyl)-*β*-cyclodextrin (HP-*β*-CD) were purchased from Sigma-Aldrich Química SA (Sintra, Portugal). Analytical grade reagents purchased from Merck (Lisboa, Portugal) were used without additional purification. Ultrapure water (Millipore Milli-Q-50 18 M*Ω* cm) was used throughout all the experiments.

### 2.2. Phase-Solubility Studies

Phase-solubility studies were performed according to the method described by Higuchi and Connors [15] and were carried out in different media, namely, deionized water, acetate buffer solutions (pH 3.5 and 5.4), and phosphate buffer solution (pH 7.4).

An excess amount of oxadiargyl (10 mg) was added to 25 mL aqueous or buffer solution containing increasing amounts of HP-*β*-CD (0–35 mmol/L). Then, the suspensions were shaken on an incubator shaker (Ika KS 4000i) at 25 ± 2°C for 24 hours. After the equilibrium was reached, the suspensions were filtered and properly diluted. The concentration of OXA was determined by spectrophotometry. Each experiment was carried out in triplicate.

The phase-solubility diagrams were obtained plotting oxadiargyl concentration versus  HP-*β*-CD concentration. The apparent stability constants, *K*_*s*_, were calculated from the phase-solubility diagrams, with the assumption of 1 : 1 stoichiometry, according to the following:

(1) $$\begin{array}{c} K_{s}=\frac{\text{slope}}{S_{0}\left(1-\text{slope}\right)}. \end{array}$$

*S*_0_ is the solubility of OXA in the absence of HP-*β*-CD.

### 2.3. Preparation of the Inclusion Complex

The oxadiargyl/HP-*β*-CD complex was prepared by kneading procedure (KN). Equimolar amounts of oxadiargyl and HP-*β*-CD were accurately weighed and mixed together using a mortar. The mixture was then triturated and an appropriate amount of ethanol was added till a homogenous paste was formed. The paste was then kneaded for 45 min and dried at 45°C in an oven under vacuum. The dried product was gently ground into a fine powder.

### 2.4. Physicochemical Characterization of OXA/HP-*β*-CD Complex

#### 2.4.1. UV-Visible Spectroscopy Experiments

Absorption spectra measurements were carried out with a Shimadzu UV-1800 PharmaSpec UV-Vis spectrophotometer. The quantification of oxadiargyl, in its free and CD-complex form, was accomplished by UV-visible spectroscopy (between 190 and 400 nm) studies. Standard curves of oxadiargyl were prepared in a mixture of methanol and water (8 : 2). 

The absorbance maximum at the wavelength of 287 nm was used to quantify oxadiargyl.

### 2.5. Nuclear Magnetic Resonance Studies

^1^H NMR data were acquired at room temperature and recorded on a Bruker Avance III operating at 400 MHz. Chemical shifts are expressed in *δ* (ppm) values relative to tetramethylsilane (TMS) as internal reference. Chemical shifts changes (Δ*δ*) were calculated according to the formula Δ*δ* = *δ*_(complex)_ − *δ*_(free)_.

NMR experiments were carried out in deuterated methanol (CD_3_OD), owing to the extremely poor aqueous solubility of OXA.

## 3. Results and Discussion

### 3.1. Phase-Solubility Studies

**Figure 2:**
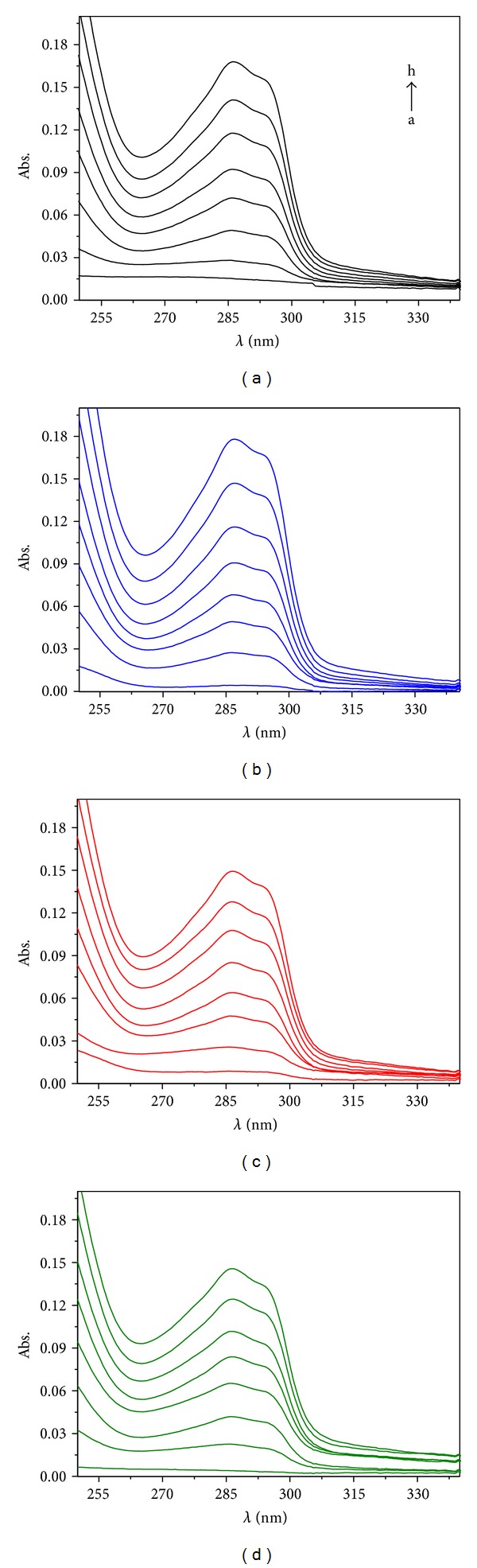
UV-Vis spectra of oxadiargyl in the presence of increasing concentrations: (a) 0; (b) 5; (c) 10; (d) 15; (e) 20; (f) 25; (g) 30; (h) 35 mM of HP-*β*-CD in (Black Line) ultrapure water, (Blue Line) acetate buffer pH 3.5, (Red Line) acetate buffer pH 5.3, and (Green Line) phosphate buffer pH 7.4.

The formation of inclusion complexes OXA/HP-*β*-CD was confirmed by UV-Vis spectrophotometry. The UV spectra of oxadiargyl in the absence and presence of increasing amounts of HP-*β*-CD and in different aqueous media (ultrapure water, acetate, and phosphate buffer electrolyte) are shown in Figure 2. Upon complexation no significant spectral changes were detected. Although the spectral band position remains practically unaltered, an increase of absorbance was observed for the UV bands of oxadiargyl spectrum upon addition of cyclodextrin, in all the different media tested (Figure 2). The observed hyperchromicity can be an evidence of the formation of OXA/HP-*β*-CD complexes either in water or in buffer solutions [16].

**Figure 3:**
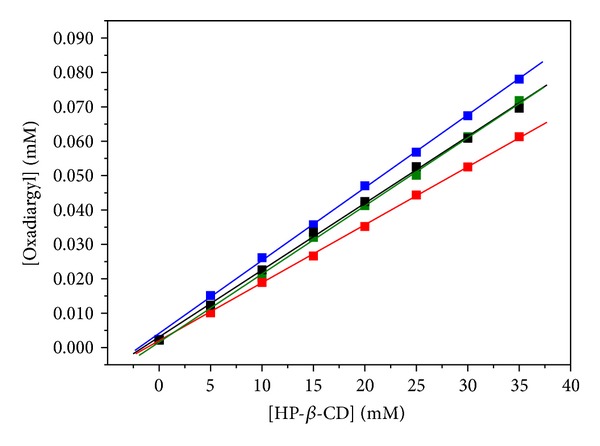
Phase-solubility diagram for oxadiargyl and increasing concentrations of HP-*β*-CD in (Black Line) ultrapure water, (Blue Line) acetate buffer pH 3.5, (Red Line) acetate buffer pH 5.3, and (Green Line) phosphate buffer pH 7.4. Each point represents the mean of three determinations.

Inclusion stoichiometry, that is, the molar ratio of oxadiargyl to HP-*β*-CD, was determined by phase-solubility study. The phase-solubility diagrams of oxadiargyl in aqueous HP-*β*-CD solutions (water and acetate and phosphate buffers) at 25°C are shown in Figure 3. As can be seen, in all cases the solubility of oxadiargyl increases linearly with the increment of HP-*β*-CD concentration. All phase-solubility diagrams of OXA with HP-*β*-CD, within the concentration range studied, displayed a typical A_L_-type diagram, according to the classification established by Higuchi and Connors [15].

Table 1 summarizes the OXA solubility, slopes, stability constants, and correlation coefficients of the phase-solubility diagrams (as a function of pH value). Results evidenced that the OXA solubility is enhanced by the presence of the macrocycle. Since the slope of the diagrams is less than 1, the complex stoichiometry was assumed to be 1 : 1 at the different aqueous media under study.

The stability constants, *K*_*s*_, of the complex were calculated from the slopes of the linear phase-solubility plots, according to the methodology described previously (see Section 2.2).

The calculated *K*_*s*_ values for OXA/HP-*β*-CD complex in ultrapure water and in buffers pH 3.5, pH 5.3, and pH 7.4 were 898 ± 25, 888 ± 13, 805 ± 17, and 886 ± 9, respectively. The stability constant and solubility of oxadiargyl complexes determined in the different aqueous media are of the same order. As a consequence of the lipophilic character of the herbicide, the solubility enhancement can be attributed uniquely to the HP-*β*-CD solubilizing ability. As the *K*_*s*_ values are high one can conclude that there is a great tendency of oxadiargyl to enter in cyclodextrin cavity and therefore the inclusion complex to be formed [17, 18].

### 3.2. Nuclear Magnetic Resonance Analysis

Nuclear magnetic resonance spectroscopy is one of the most powerful tools for elucidation and characterization of inclusion complexes [19–23]. NMR is the only technique that provides information on the orientation of the guest molecule inside the cavity and also on other important parameters related to the physicochemical characteristics of the inclusion complexes. In one-dimensional proton nuclear magnetic resonance spectroscopy (^1^H-NMR) the proton chemical shift displacements directly involved in the encapsulation process provide important information about the formation of the complexes [24]. Thus, ^1^H-NMR can provide unique structural information about stoichiometry and the inclusion of herbicide oxadiargyl into the cavity of the HP-*β*-CD. 

HP-*β*-CD cyclodextrin consists of seven identical monomers and assumes a cone-shaped structure. The hydroxyl groups and hydroxypropyl groups located on the exterior of the HP-*β*-CD contribute to its notorious aqueous solubility. The inside ring is composed of a surface of hydrophobic C-3 and C-5 hydrogens as well as glycosidic ether-like oxygen. 

**Figure 4:**
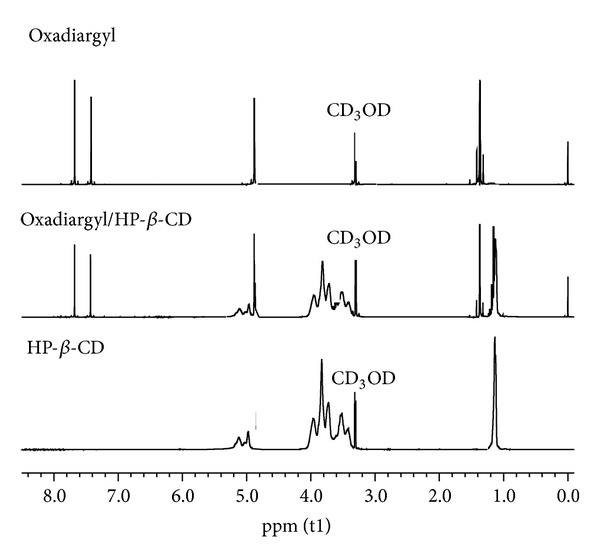
^1^H NMR spectra of OXA, HP-*β*-CD, and OXA/HP-*β*-CD inclusion complex.

^1^H-NMR spectra of OXA, HP-*β*-CD, and OXA/HP-*β*-CD are presented in Figure 4. The numbering of the hydrogen atoms faced inside and outside the CD cavity has been ascribed in accordance with the literature [25, 26]. The chemical shift displacements found in the spectra of OXA/HP-*β*-CD, when comparing with free HP-*β*-CD (host), point out the occurrence of an inclusion process. The smooth variation in chemical shifts and the absence of new peaks that could be assigned to the complex suggest the occurrence of a dynamic process with a fast exchange between free OXA and included forms.

The chemical shift (*δ*) data found for OXA, HP-*β*-CD, and OXA/HP-*β*-CD complex are shown in Table 2. Chemical shifts of the hydrogen atoms located inside the cyclodextrin cavity (H3′, H5′, and H6′) become shielded and show higher downfield shifts in the presence of oxadiargyl, whereas the hydrogen atoms outside HP-*β*-CD cavity (H1′, H2′, and H4′) present slight shifts upon complexation. In the presence of cyclodextrin the OXA protons exhibit slight upfield shifts, except for H2 aromatic proton, which indicates its interaction with the H3′ or H5′ protons of cyclodextrin and a partial inclusion of the guest inside the cavity. The significant high field shifts observed in the NMR signals of H-3′ and H-5′ of HP-*β*-CD in the inclusion complex clearly indicate the insertion of the aromatic part of the guest into the CD cavity [27, 28].

In summary, the displacements observed are indicative of the occurrence of host-guest interactions and the formation of the inclusion complex between oxadiargyl and HP-*β*-CD. The stoichiometry of complex was determined by means of the integration data between the tert-butyl protons in OXA and H1′ proton of the HP-*β*-CD [29]. The ratio of OXA/HP-*β*-CD in the inclusion complex was 1 : 1.

## 4. Conclusion

Applications of nanoscience and nanotechnology can be expected to have a significant impact on sustainable development, influencing sectors such as agrifood industry and environment. Cyclodextrins can play a major role in environmental science in terms of solubilisation and removal of organic contaminants, namely, pesticides, from soil and water.

Accordingly, the inclusion complexation behavior, characterization, and binding ability of the herbicide oxadiargyl with HP-*β*-CD were investigated. The UV-Vis data obtained clearly show an increment of oxadiargyl solubility as a function of HP-*β*-CD concentration which can be ascribed to the formation of an inclusion complex. An increment higher than 25 times was attained for OXA solubility. The complexation studies were conducted in different aqueous media and the stability constants obtained are practically of the same order. NMR studies were also accomplished and the data gathered confirm the formation of the OXA/HP-*β*-CD inclusion complex in a stoichiometric ratio of 1 : 1. 

The gathered results allow concluding that HP-*β*-CD can enhance the water solubility of oxadiargyl, a fact that can be regarded as an important step for its removal from industrial effluent and/or to increase the stabilizing action, encapsulation, and adsorption in water treatment plants.

## Figures and Tables

**Table 1:** Solubility of oxadiargyl (*S*_0_), slope, stability constant (*K*_s_), and correlation coefficient.

Medium	*S*_0_ ± SD (×10^−3^ M)	Slope	*K*_s_ ± SD (M^−1^)	*R* ^2^
Ultrapure water	0.00212 ± 3 × 10^−5^	0.0019	898 ± 25	0.999
pH 3.5	0.00237 ± 5 × 10^−5^	0.0021	888 ± 13	0.999
pH 5.3	0.00224 ± 2 × 10^−5^	0.0018	805 ± 17	0.999
pH 7.4	0.00226 ± 4 × 10^−5^	0.0020	886 ± 9	0.999

**Table 2:** ^
1^H NMR chemical shift (*δ*) data of OXA, HP-*β*-CD, and OXA/HP-*β*-CD inclusion complex.

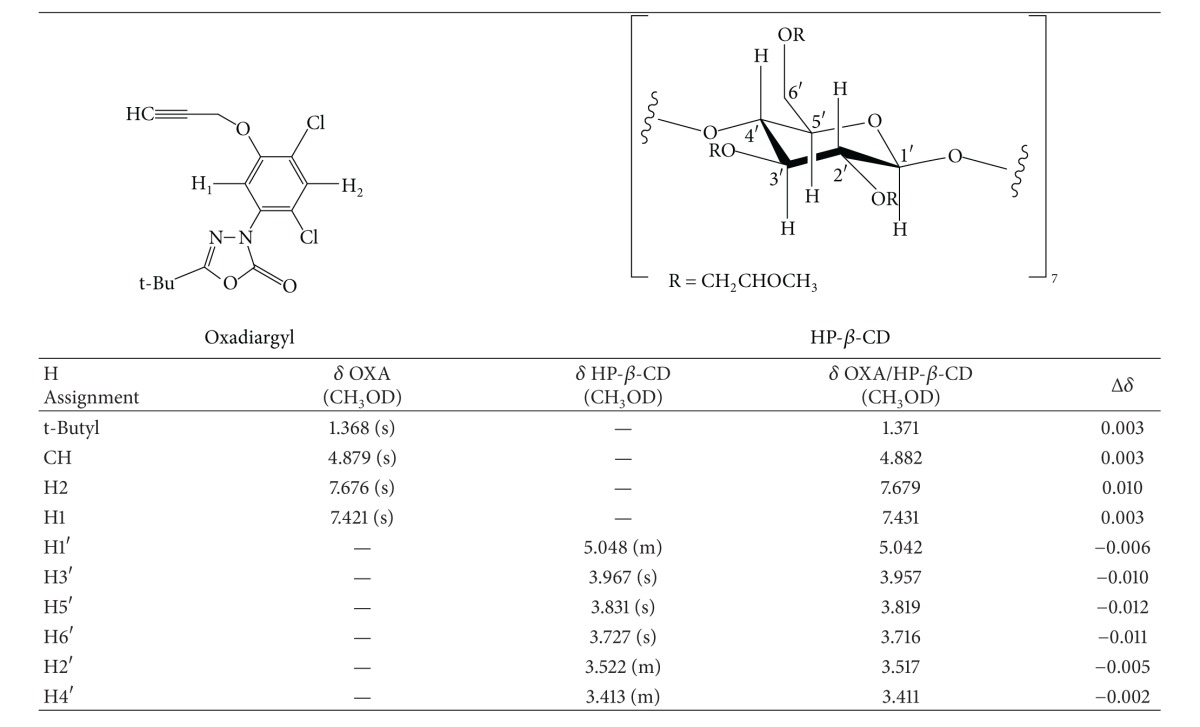

## References

[1] Zhang D, Wang X, Qiao Z-A, Tang D, Liu Y, Huo Q (2010). Synthesis and characterization of novel lanthanide(III) complexes-functionalized mesoporous silica nanoparticles as fluorescent nanomaterials. *Journal of Physical Chemistry C*.

[2] Qiu G, Huang H, Dharmarathna S, Benbow E, Stafford L, Suib SL (2011). Hydrothermal synthesis of manganese oxide nanomaterials and their catalytic and electrochemical properties. *Chemistry of Materials*.

[3] Eastburn SD, Tao BY (1994). Applications of modified cyclodextrins. *Biotechnology Advances*.

[4] Soenen SJ, Rivera-Gil P, Montenegro J-M, Parak WJ, De Smedt SC, Braeckmans K (2011). Cellular toxicity of inorganic nanoparticles: common aspects and guidelines for improved nanotoxicity evaluation. *Nano Today*.

[5] Kolkman A, Emke E, Bäuerlein PS (2013). Analysis of (functionalized) fullerenes in water samples by liquid chromatography coupled to high-resolution mass spectrometry. *Analytical Chemistry*.

[6] Karoyo AH, Sidhu P, Wilson LD, Hazendonk P (2013). Characterization and dynamic properties for the solid inclusion complexes of *β*-cyclodextrin and perfluorooctanoic acid. *The Journal of Physical Chemistry B*.

[7] Flaherty RJ, Nshime B, DeLaMarre M, DeJong S, Scott P, Lantz AW (2013). Cyclodextrins as complexation and extraction agents for pesticides from contaminated soil. *Chemosphere*.

[8] Yáñez C, Cañete-Rosales P, Castillo JP, Catalán N, Undabeytia T, Morillo E (2012). Cyclodextrin inclusion complex to improve physicochemical properties of herbicide bentazon: exploring better formulations. *PLoS ONE*.

[9] Villaverde J, Morillo E, Pérez-Martínez JI, Ginés JM, Maqueda C (2004). Preparation and characterization of inclusion complex of norflurazon and *β*-cyclodextrin to improve herbicide formulations. *Journal of Agricultural and Food Chemistry*.

[10] Villaverde J (2007). Time-dependent sorption of norflurazon in four different soils: use of *β*-cyclodextrin solutions for remediation of pesticide-contaminated soils. *Journal of Hazardous Materials*.

[11] Zhou S, Wang L, Zhang A, Lin K, Weiping L (2008). Preparation, stabilization, and bioefficacy of *β*-cyclodextrin inclusion compounds of chloramidophos. *Journal of Agricultural and Food Chemistry*.

[12] Bian H, Chen J, Cai X (2009). Inclusion complex of butachlor with *β*-cyclodextrin: characterization, solubility, and speciation-dependent adsorption. *Journal of Agricultural and Food Chemistry*.

[13] Singh M, Sharma R, Banerjee UC (2002). Biotechnological applications of cyclodextrins. *Biotechnology Advances*.

[14] Gitsopoulos TK, Froud-Williams RJ (2004). Effects of oxadiargyl on direct-seeded rice and Echinochloa crus-galli under aerobic and anaerobic conditions. *Weed Research*.

[15] Higuchi T, Connors KA (1965). Phase-solubility techniques. *Advances in Analytical Chemistry and Instrumentation*.

[16] Yáñez C, Núñez-Vergara LJ, Squella JA (2003). Differential pulse polarographic and UV-vis spectrophotometric study of inclusion complexes formed by 1,4-dihydropyridine calcium antagonists, nifedipine and nicardipine with *β*-cyclodextrin. *Electroanalysis*.

[17] Loftsson T, Hreinsdóttir D, Másson M (2005). Evaluation of cyclodextrin solubilization of drugs. *International Journal of Pharmaceutics*.

[18] Liu Y, Han B-H, Chen Y-T (2002). Molecular recognition and complexation thermodynamics of dye guest molecules by modified cyclodextrins and calixarenesulfonates. *Journal of Physical Chemistry B*.

[19] Schneider H-J, Hacket F, Rüdiger V, Ikeda H (1998). NMR studies of cyclodextrins and cyclodextrin complexes. *Chemical Reviews*.

[20] Fielding L (2000). Determination of association constants (K(a)) from solution NMR data. *Tetrahedron*.

[21] Jadhav GS, Vavia PR (2008). Physicochemical, in silico and in vivo evaluation of a danazol-*β*-cyclodextrin complex. *International Journal of Pharmaceutics*.

[22] Sinha VR, Anitha R, Ghosh S, Nanda A, Kumria R (2005). Complexation of celecoxib with *β*-cyclodextrin: characterization of the interaction in solution and in solid state. *Journal of Pharmaceutical Sciences*.

[23] Franco C, Schwingel L, Lula I, Sinisterra RD, Koester LS, Bassani VL (2009). Studies on coumestrol/*β*-cyclodextrin association: inclusion complex characterization. *International Journal of Pharmaceutics*.

[24] Cameron KS, Fielding L (2001). NMR diffusion spectroscopy as a measure of host-guest complex association constants and as a probe of complex size. *Journal of Organic Chemistry*.

[25] Yang B, Lin J, Chen Y, Liu Y (2009). Artemether/hydroxypropyl-*β*-cyclodextrin host-guest system: characterization, phase-solubility and inclusion mode. *Bioorganic and Medicinal Chemistry*.

[26] Jullian C, Morales-Montecinos J, Zapata-Torres G (2008). Characterization, phase-solubility, and molecular modeling of inclusion complex of 5-nitroindazole derivative with cyclodextrins. *Bioorganic and Medicinal Chemistry*.

[27] Ejchart A, Koźmiński W (2006). NMR of cyclodextrins and their complexes. *Cyclodextrins and Their Complexes*.

[28] Singh R, Bharti N, Madan J, Hiremath SN (2010). Characterization of cyclodextrin inclusion complexes—a review. *Journal of Pharmaceutical Science and Technology*.

[29] Chen M, Diao G, Zhang E (2006). Study of inclusion complex of *β*-cyclodextrin and nitrobenzene. *Chemosphere*.

